# 

**Published:** 1890-11-01

**Authors:** 


					TOIL
and nurses placing an undue nutritious value on such preparations aV Extract of M^t^oine" OULU Dl
made beef-tea, &c., whereas had JOHNSTON'S BOVRIL been UBed in their stead, CHEMISTS, GROCERS, &C
^ WW -fea throughout
: Wt MnM nP IH IP sasussfca the united kingdom.
^?,to your patients, to your convalescents, to those of JML ?^ your clients who have mental exertions, to use Johnsto;
tonic and a palatable food."?J. M. BEAUSOLIEL, M.D.
No. 214. Vol IX.] Saturday,
November 1st, 1890.
[One Penny.

				

